# Uncovering the Turnover Intention of Proactive Employees: The Mediating Role of Work Engagement and the Moderated Mediating Role of Job Autonomy

**DOI:** 10.3390/ijerph16050843

**Published:** 2019-03-08

**Authors:** Inyong Shin, Chang-Wook Jeung

**Affiliations:** 1 College of Business Administration, Pukyong National University, 45 Yongso-ro, Nam-gu, Busan 48513, Korea; shiny@pknu.ac.kr; 2 School of Business, Yonsei University, 50 Yonsei-ro, Seodaemun-gu, Seoul 03722, Korea

**Keywords:** proactive personality, turnover intention, work engagement, job autonomy

## Abstract

Retaining proactive employees with the potential to be high performers is recognized as an essential condition for an organization’s survival and prosperity. However, few studies have logically explained and empirically clarified the link between proactive personality, which represents a distal proactive tendency, and turnover intention to predict actual turnover behavior. With the research objective to address these research gaps, we expected that work engagement as a proximal motivational mechanism was likely to mediate the relationship between proactive personality and turnover intention, and that job autonomy as a critical job context was likely to moderate the relationship between proactive personality and work engagement. We developed a moderated mediation model incorporating these expectations. The results of the survey conducted on employees working for mid-sized manufacturing firms in Korea were consistent with our expectations. The findings of this study help uncover the intentions of turnover exhibited by proactive employees.

## 1. Introduction

In general, turnover of organization members results in the loss of human and social capital, leading to a negative impact on organizational effectiveness [1,2]. In particular, if high performers who greatly contribute to an organization leave the organization, the impact of the loss becomes much larger [3]. Since proactive employees set change-oriented goals and try to create rather than adapt to new situations [4,5], they tend to mature into high performers who control the environment surrounding them and pursue constructive change [6]. Accordingly, retaining proactive employees with the potential to be high performers is recognized as an essential condition for the survival and prosperity of an organization [7]. Thus, it is very important to discover whether or not proactive members within an organization will leave the organization and how to retain those employees.

Researchers have found that when organizational members intend to leave an organization, they tend to plan ahead rather than deciding to leave hastily [8]. According to a meta-analytic review, among the factors predicting their actual turnover behavior, the explanatory power of their intention to leave is very high [9]. Thus, turnover intention has been highlighted as an important concept. In addition, the proactivity of an employee, called proactive personality, represents the tendency to take action to change the current situation [4]. Accordingly, proactive employees’ possibility of turnover seems to be explained based on the relationship between proactive personality and turnover intention. However, few previous studies have closely examined the relationship between these two constructs. Most prior studies have only reported the correlations between them without a theoretical explanation. Inconsistent results have also been presented in the literature. For example, some scholars have found that proactive personality has a negative correlation with turnover intention (e.g., [10]), while others have indicated that proactive personality positively correlates with turnover intention (e.g., [11]). Other studies have suggested a statistically insignificant correlation between them (e.g., [12]). These discrepancies imply that the relationship between proactive personality and turnover intention might not be as straightforward as anticipated.

To address these research gaps, we believe that further research should consider the following aspects. First, it is necessary to examine processes through which proactive personality is related to turnover intention. According to the distal-proximal approach, personality as a distal trait has an effect on behavior through a proximal state such as motivation [13,14]. Thus, we need to explore a motivational construct, which provides substantive interpretations of the underlying nature of the link between proactive personality and turnover intention (cf. [15]). This leads us to focus on work engagement to identify a mechanism through which the proactive personality of employees is associated with their turnover intention because work engagement has been considered an indicator of motivation as well as well-being [16,17]. We thus develop a hypothesis that proactive personality is indirectly related to turnover intention through work engagement.

Second, the relationship between proactive personality and work engagement might vary depending on the situation. According to trait activation theory, some situations activate a specific trait more than others [18]. In particular, in motivational job design approaches, job autonomy at the core helps manifest the proactivity of job incumbents [19,20]. Thus, our attention is placed on job autonomy as a critical contextual factor. This study hypothesizes that job autonomy moderates the relationship between proactive personality and work engagement.

Lastly, in order to fully understand the relationship between proactive personality and turnover intention, it is necessary to have an integrated approach to provide a more viable explanation for the relationship. The theoretical framework builds on the job demands-resources model of work engagement, which assumes that personal and job resources independently or in combination predict work engagement, which in turn has a positive effect on work effectiveness [21,22]. In particular, the model suggests that “employees who score high on optimism, self-efficacy, resilience and self-esteem are well able to mobilize their job resources, and generally are more engaged in their work” [22] (p. 218). We adopted the model as a theoretical framework for the indirect relationship of personal resources with turnover intention through work engagement and the moderating role of job resources on this relationship. Considering that individuals with personal resources tend to try to control and impact their environments successfully [23], and individuals with proactive personality have tendency to initiate change and influence their environments [4], proactive personality seems to play a similar role as personal resources. In addition, job autonomy from job characteristics theory is one of the typical job resources [22]. Accordingly, we believe that the job demands-resources model of work engagement corresponds to our expectation that when proactive employees perceive autonomy in performing their jobs, they will be more engaged at work, and as a result, their turnover intention will be lower. We thus seek to develop a moderated mediation hypothesis that jointly accounts for job autonomy as the moderating context and work engagement as the mediating mechanism in the link between proactive personality and turnover intention.

**Figure 1:**
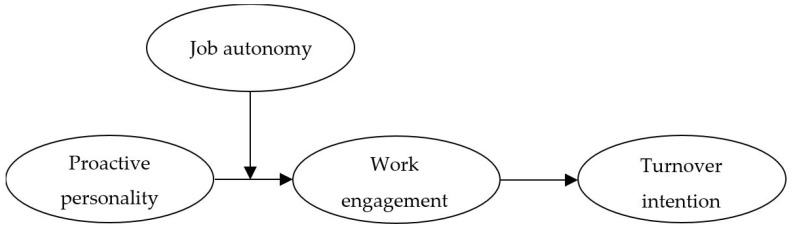
Research model.

With the considerations discussed above in mind, this study has the objectives to investigate whether work engagement mediates the link between proactive personality and turnover intention, whether job autonomy moderates the association between proactive personality and work engagement, and whether job autonomy moderates the mediated relationship between proactive personality and turnover intention via work engagement. We present our research model reflecting these objectives in Figure 1. It is expected that these systematic and comprehensive approaches will contribute to clearly understanding the turnover intention of proactive employees.

## 2. Theoretical Review and Hypothesis Development

In this section, we first explore the bridging role of work engagement in order to clearly explain the turnover intention of proactive employees. Then, by focusing on the intensifying role of job autonomy in the relationship between proactive personality and work engagement, we examine the level of engagement when proactive employees are allowed to perform their tasks autonomously. Further, we suggest the incorporated model to fully understand the roles of the psychological state and the job feature in the link between proactive personality and turnover intention.

### 2.1. Mediating Role of Work Engagement

The concept of work engagement emerged from attempts to understand the overall mental states of employees ranging from unwell-being to well-being. It was conceptualized as the opposite characteristic to burnout, which describes a state of weariness [17]. However, recent studies suggest that work engagement is not a completely opposite, positive pole of burnout, nor is it completely independent from burnout [24,25]. In other words, it has recently been recognized that work engagement has a negative relationship with burnout, but it also has its own unique aspects [24]. Therefore, in order to understand the relationships between work engagement and other constructs, it is desirable to pay attention not only to the features of work engagement overlapping with burnout, but also to its own characteristics.

Work engagement refers to “a positive, fulfilling, work-related state of mind that is characterized by vigor (high levels of energy and mental resilience while working, the willingness to invest effort in one’s work, and persistence even in the face of difficulties), dedication (a sense of significance, enthusiasm, inspiration, pride, and challenge), and absorption (being fully concentrated and deeply engrossed in one’s work, whereby time passes quickly and one has difficulties with detaching oneself from work)” [26] (pp. 74–75). To clarify how proactive personality relates to turnover intention, we draw on the distal-proximal approach, which posits that distal individual differences have indirect effects on behavior via proximal individual differences [13,14]. Since work engagement represents a motivational and mental state, we believe that it is appropriate as the proximal mechanism that accounts for the relationship between the distal tendency (i.e., proactive personality) and the work attitude (i.e., turnover intention).

We anticipate that employees with high proactive personality will be very engaged at work. Proactive personality reflects “a stable disposition to take personal initiative in a broad range of activities and situations” [27] (p. 847). Proactive employees are inclined to “scan for opportunities, show initiative, take action, and persevere until they reach closure by bringing about change” [4] (p. 105). They are also good at identifying or creating opportunities to generate favorable conditions to make them effective [28]. These characteristics of employees with proactive personality, are likely to enable them to have a vigorous, dedicated, and absorptive approach to their work and to heavily contribute to the quality of their lives in the workplace. With this in mind, scholars have asserted that “proactive personality is likely related to engagement because individuals who are involved in their work environment are also likely to immerse themselves in their work” [29] (p. 100). These findings provide strong evidence that proactive personality is significantly associated with work engagement [6,30].

We further predict that engaged workers are likely to exhibit low levels of turnover intention. Burnout has long been regarded as a stress phenomenon and a deleterious factor to increase turnover [31]. Given that work engagement and burnout are opposites, both conceptually and empirically [17], work engagement is expected to decrease turnover intention. In addition to this reasoning, it is necessary to pay attention to the unique features of work engagement, distinct from burnout. More specifically, because engaged employees are more energetic, enthusiastic, and engrossed in their work, they tend to find their work intrinsically interesting, to feel self-determined to accomplish tasks, and to revel in challenges imposed on them at work [32]. Furthermore, they tend to maintain good mental health without experiencing distress or depression [17], which would help them stay in their organizations and not intend to leave. As a result, compared to disengaged employees, engaged workers have more positive experiences in the workplace and greater attachment to their organizations, leading them to exhibit less turnover intention [33,34].

In sum, proactive employees are likely to be engaged in their work, which, in turn, is likely to reduce their intention to quit. Thus, it is reasonable to expect that proactive personality will be indirectly related to turnover intention through work engagement. Based on this expectation, we propose the following hypothesis:

**Hypothesis** **1.** *Work engagement mediates the relationship between proactive personality and turnover intention*.

### 2.2. Moderating Role of Job Autonomy

We anticipate that the strength of the relationship between the proactive personality of employees and their work engagement is likely to differ across various contexts. Trait activation theory posits that “personality traits are expressed as responses to trait relevant situational cues” [18] (p. 502). In other words, inherent traits such as personality are manifested into actions according to the situational cues related to features of the traits. Given that the job is the principal intermediary for connecting employees and organizations, it is desirable to focus on the job contexts in which they work. Further, considering that autonomy is a core job characteristic [19] and has the potential to stimulate the proactivity of employees [35], job autonomy is expected to play a role as a critical situational cue in identifying the work engagement of proactive employees.

Job autonomy is defined as “the extent to which a job allows freedom, independence, and discretion to schedule work, make decisions, and choose the methods used to perform tasks” [19] (p. 1323). Therefore, when work is designed in such a way that employees have autonomy, they are free to carry out their job tasks based on their own judgment and preferences. Those with high levels of job autonomy tend to feel more responsible for the problems that arise as they deal with their tasks and to recognize that they can sufficiently control their job outcomes [36].

Jobs with greater autonomy put fewer constraints on employees’ behavior, so job autonomy is likely to function as a weaker force that allows personality to directly drive behavior. Proactive employees are able to take advantage of autonomy as an opportunity to manage job demands [36], given that proactive personality stands for the individual tendency to identify opportunities for change and act on these opportunities [4]. Scholars have argued that “being proactive is likely most important in jobs with autonomy” [20] (p. 57).

In sum, when proactive workers have autonomous jobs, there will be synergistic effects in that they are more likely to be engaged in their work. Thus, it is expected that job autonomy will strengthen the positive relationship between proactive personality and work engagement. This expectation leads us to propose the following hypothesis:

**Hypothesis** **2.** *Job autonomy moderates the relationship between proactive personality and work engagement, such that the relationship is stronger among employees with high job autonomy than those with low job autonomy*.

### 2.3. Moderated Mediation Model

This study argues that the relationship between proactive personality and turnover intention is mediated by work engagement (Hypothesis 1) and that the link between proactive personality and work engagement is moderated by job autonomy (Hypothesis 2). By extension, these arguments represent an integrated framework in which job autonomy accentuates the motivational processes underlying the proactive personality–turnover intention relationship. That is, the rationales behind Hypotheses 1 and 2 combine to support the integration of mediation and moderation, a moderated mediation model [37,38,39]. Specifically, when proactive workers perform autonomous tasks, they are more likely to have opportunities to act in ways that are consistent with their proactivity [40], strengthening the mediating role of work engagement in accounting for the association between proactive personality and turnover intention. This expectation is based on the job demands–resources model of work engagement [21,22]. The model assumes that personal and job resources are mutually related and interact to lead to enhanced work engagement and outcomes. Personal resources refer to individuals’ sense of their ability to successfully control and influence their environment [23], which are conceptually similar to proactive personality. Job autonomy is one of the typical job resources [22]. We thus anticipate that as the autonomy of the tasks performed by proactive employees increases, their levels of work engagement will become higher and as a result their intention to deviate from the organization will be lower. This expectation leads us to propose the following hypothesis:

**Hypothesis** **3.** *Job autonomy moderates the mediated relationship between proactive personality and turnover intention via work engagement, such that the relationship is stronger among employees with high job autonomy than those with low job autonomy*.

## 3. Method

### 3.1. Data Collection Procedure and Sample Characteristics

The data used in the present study were collected from employees working in two mid-sized, for-profit organizations in the Korean manufacturing industry. Mid-sized manufacturing firms in Korea have generally faced significant turnover and resignation issues [41]. According to the Ministry of Employment and Labor of Korea, the average turnover rate in the manufacturing industry from 2013 to 2017 was 3.4% in small and medium enterprises, and 1.3% in large enterprises [42]. Therefore, it is meaningful to investigate the association of personal/job characteristics and employees’ work engagement with their intention to leave in mid-sized manufacturing firms in Korea.

A cross-sectional field survey design was adopted. Both firms had relatively small numbers of employees (i.e., 96 for firm A and 85 for firm B). Thus, the survey questionnaires were distributed to all 181 employees, rather than depending on random or convenience sampling approach. As the data collection was conducted at a single time point based on participants’ self-perception, there was a possible chance of data contamination by common method bias [43]. Following the recommendation of the prior research, in the survey questionnaire, we positioned the measurement scales of the major variables (i.e., predictor, mediator, moderator, and the final outcome variable) separated by scales of other unrelated variables to reduce any artificial inflation/deflation of correlations among items [43].

The data collection procedure is as follows. First, the purpose and theoretical/practical contributions of the current study were explained to the executives and staffs of the two firms. With coordination from the administrative staffs, a paper version of the survey questionnaire was distributed to all 181 employees in the two firms. The questionnaire included a cover page briefly describing the nature of the study as well as the issues of confidentiality and voluntary participation, followed by the scales of the psychometric properties and the questions for demographic information. In total, 170 employees participated, a response rate of 93.92%. A few missing values were imputed using the conditional mean imputation method. A multivariate outlier detection test based on Mahalanobis *D*^2^ [44] did not identify any anomalous case.

Among the 170 participants, 115 were male employees (67.65%), and the average age was 35.06 years (SD = 7.66). The average tenure in the current organization was 5.47 years (SD = 5.09) with a range of one month to 23.17 years. In terms of education level, 115 (67.65%) had a four-year bachelor’s or advanced degree. With respect to formal rank, 106 (62.35%) were in non-manager positions. One-hundred and one (59.41%) were in staffing departments, and 69 (40.59%) worked in line functions. In summary, the sample roughly represents a group of highly educated young male workers in the Korean manufacturing industry.

### 3.2. Measurement Scales

Four psychometric scales were adopted to measure the major variables. All scales were based on a five-point Likert-type scale with 1 = strongly disagree and 5 = strongly agree. Since the original scales were developed and validated in a Western context, a Korean version of the questionnaire was prepared using the translation-back-translation method [45]. With the help of two Korean–English bilinguals, a professor, and a practitioner, the items were refined in each phase of translation and back-translation to minimize loss of the validity of the original versions.

Proactive personality was measured using the abbreviated form of the Proactive Personality Scale [46]. One of six items was eliminated due to low factor loading (0.45), and the mean of the remaining five items were used in the main analysis. For the level of job autonomy, three items from Morgeson and Humphrey [19] were adopted. To measure the extent to which employees were engaged in their work, the nine items of the Utrecht Work Engagement Scale (UWES-9) [47] were used. The scale consists of three subdimensions (i.e., vigor, dedication, and absorption), and each subdimension has three items. Lastly, for turnover intention, Cammann et al.’s [48] three items were utilized. All item questionnaires as well as the values of standardized factor loading, average variance extracted (AVE), composite reliability (CR), and Cronbach’s alpha as internal consistency reliability are reported in Table 1.

The sample of the current study was collected from two separate organizations; thus, the organization (0 = firm A, 1 = firm B) was controlled. In addition, several demographic characteristics were also included as control variables to remove any spurious relationships among the major variables. According to previous studies, gender, tenure, and education may significantly influence the level of work engagement (e.g., [49,50,51]) and turnover intention (e.g., [52,53]). Therefore, gender (0 = female, 1 = male), tenure in years of working at the current organization, and education in years (e.g., 12 for a high-school graduate, 16 for a four-year university graduate) were controlled in all main analyses.

### 3.3. Data Analysis Strategy

Prior to the hypothesis testing, confirmatory factor analysis (CFA) was implemented using AMOS 24 to assess whether the measurement model appropriately fit the proposed research model. In addition, the level of data contamination by common method bias [43] as well as the discriminant, convergent validity and internal consistency reliability were examined. Descriptive statistics and correlations among the major variables were also investigated.

For each analysis of testing mediation, moderation, and moderated mediation, a two-step approach was adopted: (1) hierarchical multiple regressions were conducted using SPSS 25 (IBM, Armonk, NY, USA) [37], and (2) the results were reconfirmed by examining the 95% bootstrapped confidence intervals of the effects using PROCESS 3.2 macro for SPSS [38]. Especially for testing the moderated mediation hypothesis, a conditional process analysis was implemented using the same macro, and the index of moderated mediation (i.e., the slope of the conditional indirect effect depending on the moderator) was investigated [39].

## 4. Results

### 4.1. Measurement Model Assessment

Prior to the main analysis, CFA was conducted to examine the fit between the collected data and the hypothesized model. As presented in Table 2, the original four-factor model (i.e., Model 1) demonstrated a desirable fit (χ^2^ = 271.87, df = 161, χ^2^/df = 1.69, comparative fit index [CFI] = 0.94, Tucker-Lewis index [TLI] = 0.92, incremental fit index [IFI] = 0.94, root-mean-square error of approximation [RMSEA] = 0.06, standardized root-mean-square residual [SRMR] = 0.07), showing evidence that the measured data fit well with the proposed research model [54,55]. Additionally, possible combined factor structures were tested by aggregating the latent factors of the major variables. The chi-squared difference tests confirmed that the original four-factor model had a significantly better model fit than any other models with the aggregated factor structures (see the right column in Table 2).

Because the data in the present study were collected in a cross-sectional design and based on the participants’ self-reporting, there might be common method bias [43]. To investigate the level of data contamination, Harman’s single-factor test was conducted. The fixed single factor explained only 38.30% of the overall covariance of the major variables (i.e., lower than 50%). In addition, a CFA with a common latent factor linked to all measured items was implemented. The model fit slightly improved (*χ*^2^ = 199.61, df = 141, *χ*^2^/df = 1.42, CFI = 0.97, TLI = 0.95, IFI = 0.97, RMSEA = 0.05, SRMR = 0.06), but the overall levels of the factor loadings were not distinct from those in the original model. In summary, while there is a certain degree of data contamination by common method bias, it is not a critical threat for the main analysis for hypothesis testing.

In addition, as indicated in Table 1, Cronbach’s alpha values as the internal consistency reliabilities ranged from 0.74 to 0.92, exceeding the traditional criterion of 0.70 [56]. The AVE and CR, also presented in Table 1, are important indicators of discriminant and convergent validity [57]. Each AVE value was larger than the squared correlations between the specific variable and the related others. The CR values (0.75 to 0.95) also exceeded the “rule of thumb” criterion of 0.70. In sum, it can be concluded that the measurement model in this study has sufficient reliability and validity.

### 4.2. Descriptive Statistics and Correlations

Descriptive statistics and Pearson correlations for the four psychometric scales are shown in Table 3. Proactive personality showed a significantly positive correlation with work engagement (*r* = 0.48, *p* < 0.001) and a significantly negative correlation with turnover intention (*r* = −0.19, *p* < 0.05). Work engagement also indicated a significantly negative correlation with turnover intention (*r* = −0.47, *p* < 0.001). The relationships among the major variables support the hypothetical ideas of the present study.

### 4.3. Hypothesis Testing

Table 4 presents the results of the regression analysis to investigate mediation, moderation, and moderated mediation (the numbers in bold are relevant to hypothesis testing). Hypothesis 1 predicted that work engagement was a mediator between proactive personality and turnover intention. In Model 6 in Table 4, proactive personality showed a significantly negative main relationship with turnover intention (*β* = −0.18, *t* = −2.28, *p* < 0.05). In Model 2, proactive personality showed a significantly positive relationship with work engagement (*β* = 0.48, *t* = 6.80, *p* < 0.001). In Model 7, controlling proactive personality, work engagement showed a significantly negative relationship with turnover intention (*β* = −0.49, *t* = −6.29, *p* < 0.001), and the relationship between proactive personality and turnover intention turned out to be insignificant (*β* = 0.06, *t* = 0.71, *p* > 0.05). This is a typical pattern of full mediation based on Baron and Kenny’s [58] traditional approach.

To test the mediation effect more thoroughly, confidence intervals (CI) of indirect effect were calculated based on bootstrapping [38,39] and Monte Carlo simulation using R 3.5.1 [59]. The results are reported in Table 5. The indirect effect of work engagement based on 5000 bootstrapped resampling was significant (*b* = −0.38, 95% bias-corrected CI = [−0.56, −0.24]), whereas the direct effect was not significant (*b* = 0.09, 95% bias-corrected CI = [−0.16, 0.34]). The analysis based on the simulated distribution with 20,000 repetitions yielded an identical result (*b* = −0.37, 95% CI = [−0.55, −0.23]). In sum, the role of work engagement as a full mediator between proactive personality and turnover intention was sufficiently confirmed. Hypothesis 1 was thus supported.

Prior to testing moderation and moderated mediation, continuous independent variables were mean-centered to reduce possible multicollinearity [39,44]. In all of the regression models, the variance inflation factor (VIF) values were under 1.80, which is an appropriate level.

**Figure 2:**
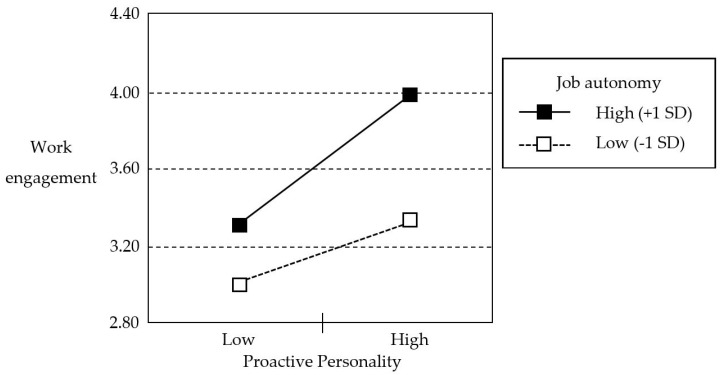
Visualization of moderation effect.

Hypothesis 2 predicted that the relationship between proactive personality and work engagement is positively moderated by the level of job autonomy. As indicated in Model 4 in Table 4, the interaction term of proactive personality and job autonomy was significant (*β* = 0.13, *t* = 2.01, *p* < 0.05). In addition, the bootstrapping test reconfirmed a significant interaction effect (*b* = 0.25, 95% bias-corrected CI = [0.0047, 0.50]) as reported in Table 6. The simple slope test [60] showed that the positive link between proactive personality and work engagement was much stronger (*b* = 0.65, *t* = 5.49, *p* < 0.001) when the level of job autonomy was high (+1 SD). When the level of job autonomy was low (−1 SD), the link was relatively weak (*b* = 0.31, *t* = 2.33, *p* < 0.05). Thus, Hypothesis 2 was supported. Figure 2 illustrates the slope difference at ±1 SD of the level of job autonomy.

Hypothesis 3 argues that the mediation effect via work engagement between proactive personality and turnover intention is moderated by the level of job autonomy. Moderated mediation is when the size/sign of an indirect effect via mediator significantly changes depending on the level of the moderator [37,38,39]. It can be concluded that there is a moderated mediation effect in the model of this study under two conditions [37]: (1) if the relationship between proactive personality and work engagement (i.e., mediator) is moderated by job autonomy (i.e., moderator), AND (2) if the relationship between work engagement (i.e., mediator) and turnover intention is still significant in the model including the interaction of work engagement (i.e., mediator) and job autonomy (i.e., moderator). The first condition was already satisfied by accepting Hypothesis 2. For the second condition, in Model 8 in Table 4, work engagement was significantly related to turnover intention (*β* = −0.49, *t* = −5.69, *p* < 0.001). Therefore, it can be concluded that there is a hypothesized moderated mediation effect.

**Figure 3:**
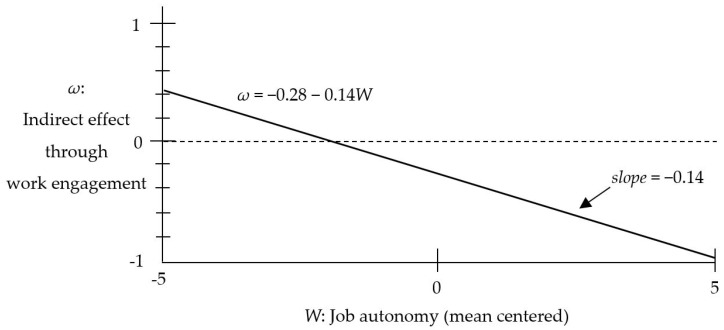
Visual presentation of the linear function of moderated mediation effect.

A conditional process analysis using PROCESS 3.2 (template number 7) was conducted to reconfirm the moderated mediation effect [38,39]. Table 7 presents the test results of the bootstrapping analysis. According to the results, the index of moderated mediation was significant (*b* = −0.14, 95% bias-corrected CI = [−0.31, −0.03]). The index of moderated mediation is the slope when the changes in the indirect effect are linearly visualized, depending on the level of the moderator, as illustrated in Figure 3 [39]. Thus, when this index (i.e., slope) is significant, the moderated mediation effect is confirmed. As hypothesized, the negative indirect effect via work engagement was more apparent (*b* = −0.37, 95% bias-corrected CI = [−0.57, −0.23]) when the level of job autonomy was high (+1 SD) than low (−1 SD; *b* = −0.18, 95% bias-corrected CI = [−0.36, −0.01]). Therefore, Hypothesis 3 was supported.

## 5. Discussion

We judged that previous research on the turnover intention of proactive employees contains two unresolved issues. First, no theoretical approach accounts for which proactive personality relates to turnover intention. Second, the empirical results for the relationship have been inconsistent. To address these research gaps, this study focused on the research model that incorporates the motivational mediator and the contextual moderator in the relationship between proactive personality and turnover intention. More specifically, this study developed the hypotheses to examine whether work engagement mediates the link between proactive personality and turnover intention, whether job autonomy moderates the association between proactive personality and work engagement, and whether job autonomy moderates the mediated relationship between proactive personality and turnover intention via work engagement. Further, using the survey data obtained from 170 employees working for mid-sized manufacturing firms in Korea, we found that work engagement plays an intermediary role in linking proactive personality with turnover intention. This study also indicated that job autonomy plays a fortifying role in explaining the positive association between proactive personality and work engagement. Finally, the results of this study showed that the indirect link between proactive personality and turnover intention through work engagement is moderated by job autonomy.

### 5.1. Theoretical Contributions and Practical Implications

Scholars broadly agree that it is significant for contemporary business organizations to have proactive employees who are likely to become high performers for their viability [3,6]. However, they have neglected to logically explain and empirically clarify the link between proactive personality, which represents individual proactive tendency, and turnover intention to predict individuals’ actual turnover. By seeking to solve these issues, this study contributes to the relevant literature.

Specifically, this study reveals that work engagement functions as a bridge that links proactive personality with turnover intention. To substantively interpret the underlying nature of the link between proactive personality and turnover intention, it is necessary to explore the intermediary mechanism that plays a mediating role in this link (cf. [15]). Drawing on the distal-proximal approach, which posits that distal dispositional tendencies indirectly relate to behaviors via proximal motivational states [13,14], we focused on work engagement because it is a motivational construct that is closer to turnover intention. Previous research has proposed work engagement as an antecedent of behavioral intention or a consequence of individual personality [29,61]. By moving beyond such research, this study underscores the importance of work engagement as a link pin in explaining the relationship between proactive personality and turnover intention. In particular, it is noteworthy that work engagement fully mediates the relationship between proactive personality and turnover intention. This finding implies that employees with proactive personality are engaged in their work, resulting in decreased levels of turnover intention. It also suggests that proactive workers with the potential to be high performers are consequently likely to benefit their organizations.

In addition, drawing on trait activation theory, which posits that traits are expressed as responses to relevant situational cues [18], we attempted to explore the job context that can potentially stimulate the proactive trait. Indeed, this study found that the relationship of the proactive dispositional tendency with the individual motivational state is contingent upon the autonomous job context. Thus, this study reaffirms the validity of the theory and broadens its applicable scope. On the other hand, the findings of this study are meaningful in that it responds to the call for research to assess the extent to which situational forces enhance or attenuate the manifestation of proactive personality [4]. By finding that high levels of job autonomy appear to enhance the positive association between proactive personality and work engagement, this study identifies job autonomy as the important boundary condition to determine the extent to which proactive personality links with work engagement. This study thus contributes to the proactivity literature in suggesting the optimal contextual factor that can harmonize with individual proactive personality.

Finally, we developed a moderated meditation model to comprehensively account for the association between proactive personality and turnover intention based on the job demands–resources model of work engagement [21,22]. We also demonstrated when proactive employees perform autonomous tasks, they are more likely to become engaged at work, which consequently leads to reducing their turnover intention. This study advances our understanding of how proactive personality relates to turnover intention and when the turnover intention of proactive employees decreases. Further, the research model of this study corresponds to Blumberg and Pringle’s [62] argument that researchers need to inclusively examine individual ability, opportunity, and motivation to understand employee behavior. In line with this argument, we found evidence consistent with the notion that the behavioral intention (i.e., turnover intention) is determined by the motivation (i.e., work engagement) that arises from the joint strength of the ability (i.e., proactive personality) and the opportunity (i.e., job autonomy).

This study also suggests practical implications for successful organizational management. Based on the findings of this study, the implications for the staff include suggesting the usefulness of selecting proactive individuals and the ways to prevent the turnover of proactive employees. The tools to assess the proactivity of applicants may be worthwhile and necessary for contemporary business organizations. Considering that this study supports that proactive personality positively relates to work engagement and indirectly, negatively relates to turnover intention, it is desirable to apply the means to identify the proactive tendencies of applicants during the selection process. After recruiting people with proactive tendencies, organizations need to pay attention to developing organizational practices, such as education and training, and creating organizational culture, such as organizational and supervisor support, that can further raise their levels of work engagement [16,29]. In particular, the degree of job autonomy is meaningful for designing job tasks and assigning them to incumbents. Accordingly, organizations should enable employees to work under autonomous working conditions that can help them maximize the benefits of their proactivity, which will minimize the turnover of employees with proactive personality.

### 5.2. Limitations and Future Research Directions

Although this study makes numerous theoretical and practical contributions, it also has several limitations to be solved in the future. Specifically, we tested our research model based on cross-sectional data, so causality of our results cannot be guaranteed. Although it is not assumed that turnover intention directly affects proactive personality and job autonomy, it is necessary to verify the research model using longitudinal data to make the findings of this study more persuasive.

This study also relied on a sample collected from two mid-sized manufacturing companies in Korea. Since Korean mid-sized business organizations have experienced frequent turnover problems [41,42], we judged that obtaining the sample from these firms is appropriate for this study. Nonetheless, it might raise concerns regarding the generalizability of our results. Thus, we anticipate that future research can replicate our research model in various settings in terms of companies, industries, and nations to greatly bolster the generalizability of our findings.

In this study, we focused on work engagement as a principal mediator to clarify the effect of proactive personality on turnover intention, given that motivation triggers behavior. However, other factors besides motivation need to be considered so that the relationship between traits and activities can be better identified. For example, considering that proactive personality contributes to network building [63] and that relational features such as network centrality are negatively related to turnover [64], relational properties are expected to play a role as another mediating path connecting proactive personality to turnover intention. Future research could consider integrating motivational and relational approaches.

## 6. Conclusions

With turbulent changes in the business environment, organizations are urged to attract people with proactive attributes and organizational members are often required to work in a proactive manner. Efforts to prevent these valuable employees from leaving are very important. This study contributes to these efforts by finding that proactive employees are first engaged in their work, and then exhibit low levels of turnover intention. In addition, when they work under autonomous working conditions, their levels of work engagement are higher and their levels of turnover intention are lower. As a result, this study suggests that in order to keep proactive workers in the organization, more attention should be given to work engagement, which is the motivation- and health-related indicator, and to job autonomy, which is a representative job characteristic.

## Figures and Tables

**Table 1:** Scale items and construct evaluation.

Construct	Item	λ	AVE	CR	α
Proactive personality	If I see something I don’t like, I fix it.	0.66	0.38	0.75	0.74
	No matter what the odds, if I believe in something I will make it happen.	0.72			
	I love being a champion for my ideas, even against others’ opposition.	0.50			
	I am always looking for better ways to do things.	0.61			
	If I believe in an idea, no obstacle will prevent me from making it happen.	0.56			
Job autonomy	I have made my own decision about how to schedule my work.	0.80	0.61	0.82	0.82
	I have made decisions about what methods I would use to complete my work.	0.85			
	I have had a chance to use my personal initiative or judgment in carrying out my work.	0.68			
Work engagement	At my work, I feel bursting with energy. (Vigor)	0.88	0.68	0.95	0.92
	At my job, I feel strong and vigorous. (Vigor)	0.91			
	When I get up in the morning, I feel like going to work. (Vigor)	0.77			
	I am enthusiastic about my job. (Dedication)	0.80			
	My job inspires me. (Dedication)	0.68			
	I am proud of the work that I do. (Dedication)	0.82			
	I feel happy when I am working intensely. (Absorption)	0.82			
	I am immersed in my work. (Absorption)	0.88			
	I get carried away when I am working. (Absorption)	0.81			
Turnover intention	I intend to look for a job outside of [company name] within the next year.	0.81	0.59	0.81	0.81
	I intend to remain with [company name]. (Reverse-coded)	0.74			
	I often think about quitting my job at [company name].	0.76			

Note: λ = standardized factor loading; AVE = average variance extracted; CR = composite reliability; α = Cronbach’s alpha. All factor loadings are significant (*p* < 0.001). The values of Cronbach’s alpha for three subdimensions of the UWES-9 are 0.88 (vigor), 0.81 (dedication), and 0.87 (absorption).

**Table 2:** Measurement model assessment.

Structure	*χ* ^2^	df	*χ*^2^/df	CFI	TLI	IFI	RMSEA	SRMR	*Δχ*^2^[*Δ*df]
Model 1: Full four factors	271.87	161	1.69	0.94	0.92	0.94	0.06	0.07	-
Model 2: Three factors PP and WE combined	351.60	164	2.14	0.89	0.87	0.89	0.08	0.08	79.73[3]
Model 3: Three factors PP and JA combined	383.64	164	2.34	0.87	0.85	0.87	0.09	0.08	111.77[3]
Model 4: Three factors JA and WE combined	398.41	164	2.43	0.86	0.84	0.87	0.09	0.08	126.54[3]
Model 5: Two factors PP, JA, and WE combined	469.23	166	2.83	0.82	0.80	0.83	0.10	0.09	197.36[5]
Model 6: Single factor All combined including TI	581.73	167	3.48	0.76	0.72	0.76	0.12	0.10	309.86[6]

Note: Second-order CFAs were conducted. PP = proactive personality, JA = job autonomy, WE = work engagement, TI = turnover intention. CFI = comparative fit index, TLI = Tucker-Lewis index, IFI = incremental fit index, RMSEA = root-mean-square error of approximation, SRMR = standardized root-mean-square residual. *Δχ*^2^ tests are relative to Model 1. All *χ*^2^ and *Δχ*^2^ are *p* < 0.001.

**Table 3:** Descriptive statistics and correlations.

Variables	Mean	SD	1	2	3	4	5	6	7
1. Organization	0.44	0.50	-						
2. Gender	0.68	0.47	−0.13	−					
3. Tenure	5.47	5.09	0.39 ***	0.10	-				
4. Education	15.48	1.96	0.23 **	−0.06	0.04	-			
5. Proactive personality	3.48	0.52	−0.12	0.17 ^*^	0.06	0.14	-		
6. Job autonomy	3.86	0.67	0.07	0.24 **	0.06	0.24 **	0.36 ***	-	
7.Work engagement	3.44	0.70	−0.07	0.10	0.07	0.00	0.48 ***	0.44 ***	-
8. Turnover intention	2.29	0.81	0.08	−0.11	−0.00	0.07	−0.19 *	−0.22 **	−0.47 ***

* *p* < 0.05, ** *p* < 0.01, *** *p* < 0.001; two-tailed tests.

**Table 4:** Hierarchical multiple regression analysis.

Variables	Work Engagement				Turnover Intention			
	Model 1	Model 2	Model 3	Model 4	Model 5	Model 6	Model 7	Model 8
Organization	−0.10	−0.01	−0.05	−0.05	0.06	0.03	0.02	0.01
Gender	0.08	0.01	−0.06	−0.04	−0.09	−0.07	−0.06	−0.02
Tenure	0.10	0.04	0.05	0.06	−0.02	0.00	0.02	0.03
Education	0.03	-0.07	−0.13	−0.15 *	0.05	0.08	0.05	0.03
PP		0.48 ***	0.37 ***	0.35 ***		−0.18 *	**0.06**	0.03
JA			0.36 ***	0.36 ***				−0.02
PP × JA				0.13 ^*^				0.07
WE							−0.49 ***	−0.49 ***
WE × JA								0.12
*R* ^2^	0.02	0.24	0.34	0.35	0.02	0.05	0.23	0.26
Adjusted *R*^2^	−0.00	0.21	0.31	0.33	−0.01	0.02	0.21	0.22
*F*	0.93	10.18 ***	13.84 ***	12.67 ***	0.77	1.67	8.32 ***	6.24 ***
*ΔR* ^2^		0.22	0.10	0.01		0.03	0.18	0.03
*ΔF*		46.19 ***	24.77 ***	4.05 *		5.20 *	39.59 ***	1.82

Note: Values are standardized regression coefficients. PP = proactive personality, JA = job autonomy, WE = work engagement, TI = turnover intention. The numbers in bold are relevant to hypothesis testing. * *p* < 0.05, ** *p* < 0.01, *** *p* < 0.001; two-tailed tests.

**Table 5:** Mediation effect of work engagement.

Bootstrapping (5000 samples)			Monte Carlo Simulation (20,000 repetitions)		
Effect	CI_low_	CI_high_	Effect	CI_low_	CI_high_
−0.38	−0.56	−0.24	−0.37	−0.55	−0.23

Note: Values are unstandardized coefficients. CI = confidence interval (95% level).

**Table 6:** Moderation effect of job autonomy.

Test of Interaction Term			Conditional Effects			
Effect	CI_low_	CI_high_	Levels	Effect	CI_low_	CI_high_
0.25	0.0047	0.50	−1 SD	0.31	0.05	0.58
			+1 SD	0.65	0.42	0.88

Note: 5000 samples. Values are unstandardized coefficients. CI = confidence interval (95% level).

**Table 7:** Moderated mediation effect of job autonomy.

Index of Moderated Mediation			Conditional Indirect Effects			
Index	CI_low_	CI_high_	Levels	Effect	CI_low_	CI_high_
−0.14	−0.31	−0.03	−1 SD	−0.18	−0.36	−0.01
			+1 SD	−0.37	−0.57	−0.23

Note: 5000 samples. Values are unstandardized coefficients. CI = confidence interval (95% level).
